# Social Determinants and Prevalence of Antenatal Depression among Women in Rural Bangladesh: A Cross-Sectional Study

**DOI:** 10.3390/ijerph20032364

**Published:** 2023-01-29

**Authors:** Nafisa Insan, Simon Forrest, Aqil Jaigirdar, Reduanul Islam, Judith Rankin

**Affiliations:** 1 Population Health Sciences Institute, Newcastle University, Newcastle Upon Tyne NE1 7RU, UK; 2 Department of Sociology, Durham University, Durham DH1 3HN, UK; 3 Maternal Aid Association, London SE1 4QG, UK; 4 Maternal Aid Association, Sylhet 3100, Bangladesh

**Keywords:** perinatal mental health, antenatal depression, Bangladesh, prevalence, social determinants

## Abstract

The prevalence of antenatal depression in Bangladesh ranges from 18 to 33%. Antenatal depression has negative impacts on the mother and child such as suicidal ideations, low birth weight, and impaired fetal development. This cross-sectional study aims to determine the prevalence and social determinants of antenatal depression in rural Sylhet, Bangladesh. Data were collected from 235 pregnant women between March and November 2021. The validated Bangla Edinburgh Postnatal Depression Scale was used to measure antenatal depressive symptoms (ADS). Background information was collected using a structured questionnaire including the Duke Social Support and Stress Scale, pregnancy choices, and WHO Intimate Partner Violence questions. Point-prevalence of antenatal depression was 56%. Intimate partner violence (IPV) before pregnancy (adjusted odds ratio (AOR) 10.4 [95% confidence interval (CI) 2.7–39.7]) and perceived husband’s male gender preference (AOR 9.9 [95% CI 1.6–59.6]) were significantly associated with increased odds of ADS among pregnant women. Increased family support was a significant protective factor for ADS (AOR 0.94 [95% CI 0.91–0.97]). Antenatal depression commonly occurs in rural Sylhet, Bangladesh, highlighting the need for improved screening and management within these settings. The findings suggest the need for community-based interventions for women with low family support and experiencing intimate partner violence, and educational programs and gender policies to tackle gender inequalities.

## 1. Introduction

The Global Burden of Disease Study in 2017 found depression to have prevailed as a leading cause of non-fatal health loss for almost three decades [1], with women being about twice as likely than men to develop depression in their lifetime [2]. Although pregnancy is mostly viewed as a positive experience, women have increased vulnerability to depression during and immediately after pregnancy [3]. The literature mostly focuses on the postnatal period, whereas evidence suggests that two-thirds of postnatal depression actually presents antenatally [4,5], making the antenatal period a key phase to target with screening and interventions. Antenatal depression, symptomized by persistent low mood, crying, loss of appetite, loss of sleep, and sadness, among other symptoms, has negative consequences on the mother and child such as suicidal ideations, low birth weight, and impaired fetal development [6].

Bangladesh is one of the most densely populated countries and is listed as a low–middle income country (LMIC) by the World Bank [7]. The prevalence of antenatal depression in Bangladesh has been found to range between 18 and 33% [3,8], which is higher than worldwide estimates of 10–20% [9]. This may be due to the lack of mental healthcare integration into antenatal healthcare, poor understanding and stigma surrounding mental health conditions, and higher socioeconomic deprivation [10]. Despite this burden, mental health remains a neglected topic within the Bangladeshi population, government, and health system. Only 0.5% of the total health budget is allocated to mental health [11]. This is a small proportion considering the burden attributable to psychiatric disorders in Bangladesh is approximately 11.2% [12]. Furthermore, there is only one mental health institute in Bangladesh, the National Institute of Mental Health (NIMH), and limited mental health hospitals, the majority of which are located in urban areas with the density of psychiatric beds in and around the capital Dhaka being five times higher than anywhere else [12]. This raises a problem as 70% of the population live in rural areas. These areas are often served by informal providers and non-governmental organisations, such as the Maternal Aid Association (MAA) [12]. This creates limited perinatal mental healthcare services for pregnant women in rural regions of Bangladesh.

Previous studies in Bangladesh have found certain social determinants associated with antenatal depression. Social determinants are considered to be mostly responsible for health inequalities; hence, this study focuses on such determinants rather than obstetric risk factors, especially given the social and cultural dynamics within antenatal depression. Both Nasreen et al. [3] and Gausia et al. [8] found intimate partner violence (IPV) and low family support to be significantly associated with antenatal depression in pregnant women in rural Bangladesh. Gausia et al. [8] also found male gender preference to play an important role in antenatal depression. It is important to investigate these social determinants of antenatal depression, especially in a country such as Bangladesh which often neglects these issues, to understand and tackle the underlying mechanisms that play a role in antenatal depression. These previous studies [3,8] were both conducted in the capital of Bangladesh, Dhaka. There are no known studies conducted in the Sylhet division, which has the highest fertility rate and lowest contraceptive use [13,14]. Contraceptive prevalence rate is only 4.8% in Sylhet compared to the national average of 62.4% [15]. This is a concern as high fertility rates and low contraceptive rates highlight lack of education, family planning services, and gender equity within this region. It is essential to carry out studies in Sylhet to understand these social determinants and how they contribute to antenatal depression. Therefore, the aim of this study was to determine the prevalence and social determinants of antenatal depression among women in rural Sylhet, Bangladesh.

## 2. Materials and Methods

### 2.1. Study Design, Setting, and Sampling

**Figure 1:**
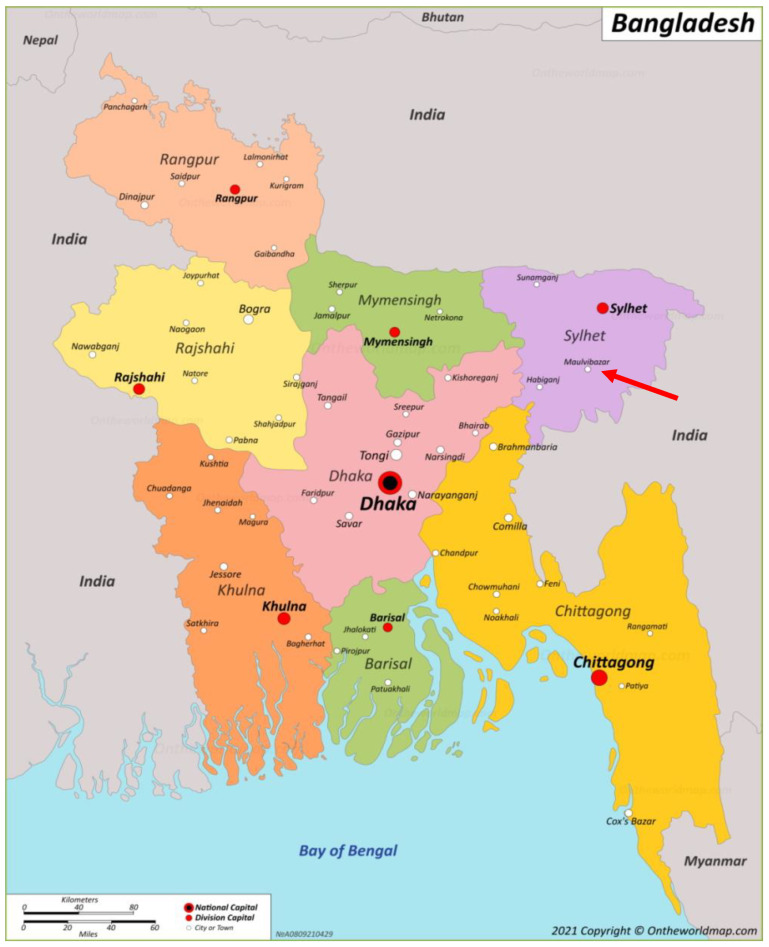
Map of Bangladesh [17] showing divisions and districts (red arrow pointing to study location).

This study followed a cross-sectional survey design to estimate a point-prevalence of antenatal depression and analyse the associated social determinants. The study setting was MAA health camps in Moulvibazar. Moulvibazar is a town in the Moulvibazar district within the Sylhet division located in north-eastern Bangladesh (Figure 1) with a population of approximately 41,358. Pregnant women attending and registering for the health camps were the study population and sampling frame used for this study. Convenience sampling was used to recruit participants, whereby pregnant women aged 18 or older in any trimester of their pregnancy attending the MAA health camps were eligible to take part in the survey with informed consent. Women with a known pre-existing diagnosis of any mental health condition were excluded as this would skew the measurement of antenatal depression, creating measurement bias. Women below the age of 18 were excluded due to ethical concerns. Using the sample size for prevalence studies calculation [16], a sample size of 227 was calculated to be adequate to estimate the prevalence of antenatal depression.

### 2.2. Survey Instrument

The survey consisted of five sections: (a) general background information, (b) Duke Social Support and Stress Scale (DUSOCS), (c) pregnancy choices, (d) Edinburgh Postnatal Depression Scale—Bangla (EPDS-B), and (e) WHO IPV. The survey was first written in English to develop the constructs and items. The English version was sent to experts in global health, maternal health, and sociology who have experience and knowledge on data collection, particularly in LMICs within South Asia. The experts provided feedback on the design, clarity, and content of the survey, providing initial face and content validity.

A systematic cultural adaptation process based on Khan and Avan [18] was used to develop, translate, and validate the survey. This involved six steps and aimed to ensure that each item was assessing the construct it was attempting to understand. Each item was first written in English, and the process of adaptation was (1) translation into Bangla independently by NI and RI; (2) comparing these translations, assessing technical equivalence of these, and then producing final translations by consensus for testing; (3) field research with project staff and mothers of young children to test understanding of the translations and to improve them; (4) finalisation of the tool for pre-testing; (5) pre-testing in the community to assess usability; and (6) assessor training and pilot-testing.

### 2.3. Dependent/Outcome Variable—Antenatal Depressive Symptoms (ADS)

ADS was measured using the EPDS-B. The EPDS is a self-report questionnaire consisting of ten items, each with four possible responses related to the subject’s mood or feelings in the last seven days [19]. Although originally meant for screening of postnatal depression, studies have shown it to be just as appropriate for use in the antenatal period [20]. Each item is scored between 0 and 3, with a total EPDS score ranging between 0 and 30. The Bangla translated version of the original EPDS has been validated in a Bangladeshi setting [19]. The validation study on EPDS-B against standard diagnosis of depression using the Structured Clinical Interview (SCID) for DSM-IV among Bangladeshi women showed a sensitivity of 89% and a specificity of 87% at cut-off scores of 10 [19]. Therefore, pregnant women scoring 10 or above in the EPDS-B were categorised as having ADS and pregnant women scoring less than 10 were categorised as non-ADS. The EPDS-B has a Cronbach’s alpha of 0.84, suggesting good internal consistency [19].

### 2.4. Independent Variables—Social Determinants

General background information such as trimester of pregnancy, age, marital status, highest education level, incoming-earning activity, and husband’s employment were collected in section A of the survey.

Section B consisted of the social support section of the DUSOCS, which captures an individual’s perceptions of how supportive their relationships are [21]. It also allows for the identification of one’s most supportive relationship. The DUSOCS creates six scores: family support, family stress, non-family support, non-family stress, total social support, and total social stress. For this study, we were interested in family support. A 3-point scale was used (“none = 0”, “some = 1”, and “a lot = 2”) in which the pregnant women rate their family members as people who give personal support (6 items). The DUSOCS was slightly adapted, as recommended by the literature, for this study in order to capture all the important relationships for pregnant women in rural areas of Bangladesh [22]. All scores range from 0 to 100, with higher scores indicating higher levels of support. The family support items has a test-retest reliability of 0.76 and Cronbach’s alpha of 0.7. This suggests excellent reliability [21].

Section C entailed questions around certain pregnancy choices: gender preference of both mother and father, unwanted pregnancies, and fear of childbirth.

The final section consisted of questions relating to IPV. These questions covered the three main areas of violence as defined by the WHO: verbal, physical, and sexual violence [23]. A 4-point scale was used (“no = 0”, “yes, once = 1”, “yes, a few times = 2”, and “yes, many times = 3”) in which pregnant women rate whether (and how often) they experienced each type of IPV both before their pregnancy and during the current pregnancy. The responses were coded to “yes” or “no” for all forms of IPV before pregnancy and during pregnancy to create 2 separate variables (IPV before and IPV during pregnancy) due to the small sample sizes for some of the 4-point responses for individual types of violence.

### 2.5. Data Collection Procedure

Data were collected by MAA researchers in the health camps and the new clinic in Moulvibazar, Sylhet, from 10 March 2021 to 2 November 2021. Pilot data collection using the survey from 36 pregnant women was conducted on 10 March 2021 in the Moulvibazaar health camp, followed by a meeting with the MAA researchers to gain feedback on the clarity, design, and content of the survey within the target population. No changes to the survey were deemed necessary by the MAA team; therefore, data from the pilot phase were included in the final sample and data collection was resumed. 

Information regarding the study were verbally administered to the participants in Bengali and both written and verbal informed consent was acquired. Survey interviews were conducted in a private area of the health camps/clinic and care was taken to ensure participants were comfortable whilst answering the questions. The interviews lasted on average 15 minutes.

### 2.6. Statistical Analysis

Data from Excel were exported into the Statistical Package for the Social Sciences (SPSS) for coding and analysis. Categories were created from the total EPDS-B scores to indicate those with EPDS-B scores of 10 or more to have ADS and those with lower than 10 to be non-ADS. Means and standard deviations were used to describe normally distributed continuous data, and median and interquartile ranges (IQR) were used for non-normally distributed continuous data. Frequencies and percentages were used to describe categorical variables. 

An independent samples t-test was used to compare means between groups. Bivariate analyses (chi-squared and Fisher’s exact) were conducted between each independent variable and the outcome variable of ADS and non-ADS independently to identify possible determinants that were associated with ADS. The minimum sample size for binary logistic regression was calculated to be 227 using the pmsampsize package on STATA as recommended by Riley et al. 2020 [24]. Therefore, the use of a logistic regression model was justified for this study. The independent variables that were significantly associated with ADS in the bivariate analyses were considered as possible social determinants and were entered into a multivariate logistic regression model, and adjusted odds ratios (AOR) and 95% confidence intervals (CI) were produced. The minimum variance inflation factors (VIFs) from the regression suggested no severe multicollinearity between the independent variables.

### 2.7. Ethical Considerations

This study was approved by the Faculty of Medical Sciences Research Ethics Committee, part of Newcastle University’s Research Ethics Committee, ref 2049/4634/2020. This committee contains members who are internal to the faculty but independent to the study. This study was reviewed by members of the committee, who must provide impartial advice and avoid significant conflicts of interest. The study was also approved by the Sylhet Women’s Medical College Research Ethics Board in Bangladesh.

## 3. Results

Overall, there were 235 respondents with ages ranging from 18 to 38 years and a median age of 25. The pregnant women were in varying stages of their pregnancy, with 100 women (42.6%) in their second trimester, 71 women (30.3%) in their third trimester, and 57 women (24.2%) in their first trimester. Seven women did not know which trimester they were in. All the respondents were married, with the majority (75.8%) having no education or having ended their education before completing class 10, i.e., having not gained their Secondary School Certification (SSC) qualifications (equivalent to GCSE in the UK). The remaining had some level of education SSC and above. The majority of respondents, 97.9%, do not undertake any paid work, with the husband being the sole breadwinner for the family through work such as fishing, farming, construction, or business (96.6% of respondents’ husbands undertake paid work). See Table 1 for a full summary of respondents’ characteristics.

The majority of the respondents, 207 (88.1%), did not know the gender of the baby they were having. Nine women (3.8%) stated they were having a boy and seven (3.0%) stated they were having a girl. The rest of the women, 12 (5.1%), preferred not to say what gender of the baby they were having. The majority of the women, 133 (56.6%), stated they had no preference for the gender of the baby and the majority, 119 (50.6%), perceived their husband to also have no preference. Almost a quarter of the women, 51 (21.7%), stated they desired to have a boy compared to 31 (13.2%) who wanted a girl, and 65 (27.7%) perceived their husband’s preference to be a son compared to 30 (12.8%) who perceived their husband to want a girl. 

Most pregnant women, 142 (60.4%), stated that their pregnancy was wanted at this time compared to 20 (8.5%) who stated they did not want the pregnancy. Similarly, 152 (64.7%) pregnant women perceived their husband to also want the pregnancy compared to 17 (7.2%) who felt their husband did not want the baby at this time. The remaining 73 (31.1%) pregnant women preferred not to say whether the pregnancy was wanted or not and 66 (28.1%) preferred not to say whether they felt their husband wanted the baby or not. A total of 200 (85.1%) pregnant women were worried about childbirth/labour compared to 17 (7.2%) who stated they were not worried. The remaining 18 (7.7%) preferred not to say. 

From the DUSOCS results, the family support score was used for the analysis, which ranged from 7.1 to 100. The mean family support score was 50.0 (SD 17.3). In this study, only 12.3% and 12.8% of pregnant women stated they receive no support from their husband and in-laws, respectively. The remaining receive some (45.5% and 66.8%) or a lot (42.1% and 20.4%) of support from their husband and in-laws, respectively.

From the IPV questions, 64 (27.2%) pregnant women stated they have experienced IPV before their current pregnancy compared to 86 (36.6%) who stated they have not experienced IPV before their current pregnancy. Furthermore, 110 (46.8%) stated they have experienced IPV during their current pregnancy compared to 32 (13.6%) who stated they have not experienced IPV during their current pregnancy. 

**Figure 2:**
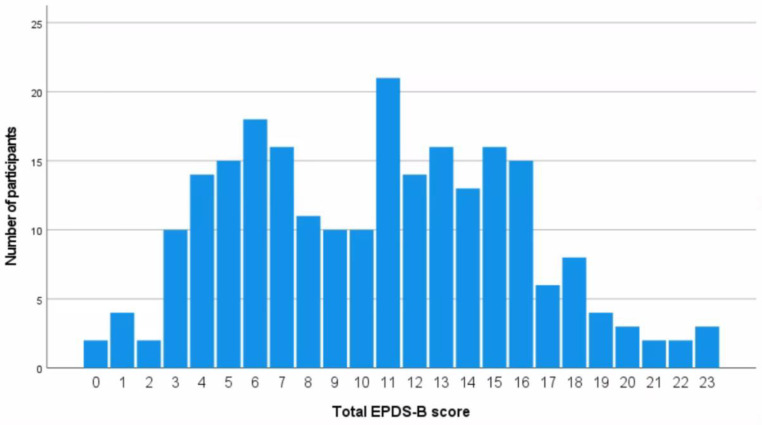
Bar graph showing the distribution of total EDPS-B scores.

Of the 235 respondents, 133 (56.6%) had total EPDS-B scores of 10 or above, indicating a point-prevalence of antenatal depression of 56.6% (95.5% CI 50.0–63.0%). Figure 2 shows the distribution of total EPDS-B scores among respondents, which ranged from 0 to 23. The mean EPDS-B score among all respondents was 10.5 (SD 5.2). The mean EPDS-B score for respondents without ADS and those with were 5.6 (SD 2.2) and 14.3 (SD 3.2), respectively. The difference of −8.8 (95% CI −9.5–−8.0) in the mean scores was statistically significant (t = −23.7, df = 233, *p* < 0.001), indicating those with depressive symptoms had significantly higher EPDS-B scores than those without depressive symptoms.

A number of independent potential social determinants for antenatal depression were examined in this study. From the bivariate analyses, IPV before pregnancy, IPV during pregnancy, pregnant woman’s gender preference, perceived husband’s gender preference for the baby, and family support were significantly associated with ADS (Table 2).

After performing the multivariate logistic regression (Table 3), IPV before pregnancy, family support, and perceived husband’s gender preference were found to be significant determinants. Pregnant women who have experienced IPV before their pregnancy had 10 times higher odds of ADS compared to pregnant women who have not experienced IPV before pregnancy (AOR 10.4 95% CI 2.7–39.7). ADS was significantly associated with low family support, whereby as family support increases, the odds of ADS decrease (AOR 0.94 [95% CI 0.91–0.97]). Pregnant women who perceived their husbands to want a son had almost 10 times higher odds of antenatal depression compared to pregnant women who perceived their husband to have no gender preference (AOR 9.9 [95% CI 1.6–59.6]).

## 4. Discussion

### 4.1. Key Findings

This study aimed to determine the prevalence and identify social determinants of antenatal depression in rural Sylhet, Bangladesh. This study found a point-prevalence of antenatal depression of 56.6%. Low family support was found to be a significant determinant of ADS, while perceived husband’s male gender preference and IPV were found to significantly increase the odds of ADS.

The prevalence of antenatal depression determined in this study is higher than previous studies conducted by Gausia et al. [8] and Nasreen et al. [3], which found a prevalence of antenatal depression in rural Bangladesh of 33% and 18.3%, respectively. A higher prevalence found in this current study may be due to methodological differences such as study location and population. Previous studies were conducted in Dhaka, the capital of Bangladesh, whereas our study was conducted in the division and district of Sylhet, which is a rural area. The Sylhet division has the highest fertility rate and lowest contraceptive use [13,14], with a contraceptive prevalence rate of 4.8% in Sylhet compared to the national average of 62.4% [15]. This is a concern as high fertility rates and low contraceptive rates highlight lack of education, family planning services, and gender equity within this region. Furthermore, the Sylhet region has geographical characteristics such as hilly areas, which make it difficult for universal health coverage in Bangladesh [15]. This is reflected by the 25.7% difference in antenatal care coverage between Khulna (second largest of the eight divisions in Bangladesh) and Sylhet, with Sylhet having low levels of antenatal care [15]. Therefore, these differences may play a role in the higher prevalence of antenatal depression in our study compared to previous studies. Nevertheless, differences may also be due to smaller sample size in our study, which could affect study power.

The association between antenatal depression and low family support is reflected in many previous studies [3,5,8,25,26]. Family support is of special importance in Bangladeshi communities, where women often live with their in-laws after marriage [3]. Therefore, family dynamics play a key role in a pregnant woman’s emotional well-being and support from her newly formed family, especially her husband and mother-in-law, is essential in making her feel accepted and protected during her pregnancy. Furthermore, women are expected to carry out the household chores, which can become more difficult during pregnancy. Therefore, practical support, as well as emotional support, from family members during this time can be a source of ease, preventing depressive symptoms. Practical support can be displayed through helping with household chores such as cooking, washing, and cleaning. This type of support may also be seen as a sign of approval and acceptance from the in-laws, which can have positive impacts on the mental health of pregnant women [3].

IPV has been well established in the literature as an important social determinant of antenatal depression [27]. IPV experienced before pregnancy was significantly associated with ADS and was identified to be the most important social determinant of ADS in this study. However, IPV experienced during pregnancy, although showing increased odds of ADS, was not statistically significant. Nasreen et al. [3] and Gausia et al. [8] both found that IPV during pregnancy was significantly associated with ADS in Bangladesh. Therefore, it would not be appropriate to conclude that IPV during pregnancy is not associated with ADS in this study as the non-significance may be due to the small sample size of the IPV during pregnancy sub-groups, which may be underpowered [28]. Pregnant women undergo social, emotional, and physical isolation and pressure when experiencing IPV, which is heightened during pregnancy due to worries about the unborn baby’s health [29]. Further studies with larger sample sizes are required to improve the power related to sub-group analyses.

The husband’s gender preference for the baby as perceived by the pregnant woman has not been widely investigated in Bangladesh. A study of pregnant women in Iran [30] found that the husband’s preference for a male child is significantly associated with antenatal depression in women. Similarly, a study of pregnant women in China [31] found that no gender preference in the spouse was associated with a significant reduction in persistent depression. These findings are also reflected in our study, whereby pregnant women who perceived their husband to want a son compared to having no gender preference have higher odds of antenatal depression. However, there was no significant association between the woman’s own gender preference and antenatal depression, which is contradictory to many previous studies [8,28,32,33,34]. Male gender preference is a common phenomenon in Bangladeshi communities and has been associated with antenatal depression [26]. This again reflects the importance of the husband in decision making and power dynamics in Bangladeshi families, whereby the husband’s preference is taken to be more important. A husband’s desire for a son may create stress for many pregnant women if they are unable to give him a son, which can result in serious repercussions. This finding demonstrates the need for more whole-family interventions as a pregnant woman’s family, in particular her husband, plays a key role in her mental well-being.

These social determinants identified within this study are complex and interlinked factors. Socially constructed gender roles favour and elevate the status of men, creating gender inequalities in Bangladesh [35]. These gender inequalities can be displayed through IPV where men feel they have power over women, pressure on pregnant women from her husband or in-laws to give birth to a son who can provide for the family in the future, and lack of family support during pregnancy. Stigma around mental health and women’s health also prevents women from speaking up about violence, family issues, and their emotions, resulting in many pregnant women feeling a lack of support and being vulnerable to depressive symptoms during their pregnancy [36].

### 4.2. Strengths and Limitations

This study has included a widely used and validated scale to measure antenatal depression in pregnant women in Bangladesh [19]. This increases the accuracy of measuring antenatal depression and allows for effective comparisons to be made with previous studies. Another strength of this study is that the survey was reviewed by experts and piloted within the population of interest, which allowed us to test the suitability and appropriateness of the survey in that setting and allowed the MAA volunteers to gain training in delivering the survey. 

Furthermore, considering the likely low levels of literacy in rural Bangladesh, the survey was delivered by trained MAA volunteers in Bengali and not self-administered. This ensured that pregnant women clearly understood the survey as there was someone there to explain the instructions and questions to them. Nevertheless, this may introduce some response bias as pregnant women may have provided responses that they felt were socially acceptable or not provided honest answers to sensitive questions due to the limited anonymity. Women may feel scared or judged whilst answering questions regarding IPV, pregnancy choices, and EPDS-B items, especially if their family members were present around them. This was highlighted by the MAA researchers after the pilot surveys who found that women were less likely or hesitant to answer questions related to IPV if they came to the health camps with their family. This is also evident from the high proportion of ‘prefer not to say’ responses. Response bias may have led to an under-reporting of certain social determinants and ADS. To minimise this bias, MAA volunteers reassured pregnant women that their answers would remain anonymous and confidential, and they built a good rapport with the women beforehand. 

As the study was cross-sectional, temporality and causality cannot be established as both the outcome and social determinants were measured at the same time. However, this study design was still deemed appropriate and addresses the objective of identifying the social determinants which have an association with antenatal depression in Bangladesh, which can be used to recommend the development of tailored interventions. Nevertheless, given the small sample size, the results cannot be generalizable to the whole population. However, the results are effective in providing an initial insight into the social determinants of antenatal depression in rural Sylhet, Bangladesh, upon which further research can be conducted.

## 5. Conclusions

The findings from this study suggest a high prevalence of antenatal depression in rural Sylhet, Bangladesh, highlighting the need for interventions and policies to tackle such high rates. IPV, low family support, and perceived husband’s male gender preference were found to be associated with antenatal depression. This highlights the need for more tailored interventions for pregnant women with low family support or those facing IPV, ensuring they are receiving the support they need and educating families on pregnancy support. There is also a need for educational programs and gender policies to tackle gender inequalities within these communities. Further research should include qualitative in-depth interviews with pregnant women and their families to understand the mechanisms within these social determinants.

## Figures and Tables

**Table 1:** Summary of respondent (pregnant women) characteristics.

Variable	Study Sample (*n* = 235)
**Age**	
Median age in years, (IQR)	25 (6)
**Trimester, *n* (%)**	
First	57 (24.2)
Second	100 (42.6)
Third	71 (30.3)
Unknown	7 (3.0)
**Marital status, *n* (%)**	
Married	235 (100)
**Highest level of education, *n* (%)**	
No schooling/Pre-SSC	178 (75.8)
SSC or above	56 (23.8)
Do not know	1 (0.4)
**Paid work, *n* (%)**	
Yes	5 (2.1)
No	230 (97.9)
**Gender of baby, *n* (%)**	
Girl	7 (3.0)
Boy	9 (3.8)
Do not know	207 (88.1)
Prefer not to say	12 (5.1)
**Gender preference, *n* (%)**	
Girl	31 (13.2)
Boy	51 (21.7)
No preference	133 (56.6)
Prefer not to say	20 (8.5)
**Perceived husband’s gender preference, *n* (%)**	
Girl	30 (12.8)
Boy	65 (27.7)
No preference	119 (50.6)
Prefer not to say	21 (8.9)
**Want pregnancy, *n* (%)**	
Yes	142 (60.4)
No	20 (8.5)
Prefer not to say	73 (31.1)
**Perceive husband wants pregnancy, *n* (%)**	
Yes	152 (64.7)
No	17 (7.2)
Prefer not to say	66 (28.1)
**Worried about childbirth/labour, *n* (%)**	
Yes	200 (85.1)
No	17 (7.2)
Prefer not to say	18 (7.7)
**Family support**	
Family support score, mean (SD)	50.0 (17.3)
**Intimate Partner Violence (IPV), *n* (%)**	
**Before pregnancy**	
Yes	64 (27.2)
No	86 (36.6)
Prefer not to say	85 (36.2)
**During pregnancy**	
Yes	32 (13.6)
No	110 (46.8)
Prefer not to say	93 (39.6)
**Antenatal Depressive Symptoms (ADS), *n* (%)**	
Yes	133 (56.6)
No	102 (43.4)

IQR = Interquartile Range; SD = standard deviation.

**Table 2:** Bivariate analysis of social determinants with antenatal depressive symptoms among pregnant women in MAA health camps in rural Sylhet.

Variables	ADS *n* = 133 (%)	Non-ADS *n* = 102 (%)	*p*-Value ^c^
**Trimester**			
First	33 (24.8)	24 (23.5)	
Second	56 (42.1)	44 (43.1)	0.79
Third	37 (27.8)	34 (33.3)	
**Education**			
No schooling/Pre-SSC	106 (79.7)	72 (70.6)	0.084
SSC or above	26 (19.5)	30 (29.4)	
**IPV before pregnancy**			
Yes	53 (39.8)	11 (10.8)	<0.001 *
No	37 (27.8)	49 (48.0)	
**IPV during pregnancy**			
Yes	26 (19.5)	6 (5.88)	0.002 *
No	56 (42.1)	54 (52.9)	
**Gender preference**			
Boy	38 (28.6)	13 (12.7)	
Girl	15 (11.3)	16 (15.7)	0.013 *
No preference	69 (51.9)	64 (62.7)	
**Perceived husband’s gender preference**			
Boy	51 (38.3)	14 (13.7)	
Girl	15 (11.3)	15 (14.7)	<0.001 *
No preference	57 (42.9)	62 (60.8)	
**Want baby**			
Yes	89 (66.9)	53 (52.0)	0.524
No	14 (10.5)	6 (5.9)	
**Perceive husband wants pregnancy**			
Yes	99 (74.4)	53 (52.0)	0.606
No	10 (7.5)	7 (6.9)	
**Variables**	**ADS mean (SD)**	**Non-ADS mean (SD)**	***p*-value ^t^**
**Age**	24.33 (5.43)	24.23 (4.20)	0.871
**Family support**	46.4 (18.7)	52.3 (14.7)	0.009 *

^c^ = chi-square test for association or Fisher’s exact test. ^t^ = independent samples *t*-test. * = statistical significance at 0.05 level. SD = standard deviation. Percentages do not add to 100 as an answer of ‘prefer not to say’ was not included in the analyses. SSC = Secondary School Certification; ADS = antenatal depressive symptoms.

**Table 3:** Multivariate logistic regression model showing associated factors of antenatal depressive symptoms among pregnant women in MAA health camps in rural Sylhet.

Variables	AOR (95% CI)	*p*-Value ^c^
**IPV before pregnancy**		
Yes	10.4 (2.7–39.7)	<0.001 *
No	1	
**IPV during pregnancy**		
Yes	1.4 (0.3–7.1)	0.656
No	1	
**Gender preference**		
Boy	0.21 (0.04–1.2)	0.085
Girl	0.34 (0.04–3.2)	0.348
No preference	1	
**Perceived husband’s gender preference**		
Boy	9.9 (1.6–59.6)	0.012 *
Girl	2.4 (0.29–19.1)	0.418
No preference	1	
**Variables**		***p*-value ^t^**
**Family support**	0.94 (0.91–0.97)	<0.001 *

^c^ = chi-square test for association or Fisher’s exact test. ^t^ = independent samples *t*-test. AOR = adjusted odds ratio. * = statistical significance at 0.05 level. SD = standard deviation. CI = confidence interval.

## Data Availability

The datasets used and analysed during the current study are available from the corresponding author upon reasonable request.
